# An operational key performance indicator framework and reference data from a multi-room pencil beam scanning proton therapy center

**DOI:** 10.1371/journal.pone.0360926

**Published:** 2026-10-14

**Authors:** Jatinder Saini, Tony Wong, Marco Schwarz, Randall McClure, Annemarie Shepherd, Jing Zeng, Ramesh Rengan

**Affiliations:** 1 Department of Radiation Oncology, University of Washington School of Medicine, Seattle, Washington, United States of America; 2 Fred Hutchinson Cancer Center-Proton Therapy, Seattle, Washington, United States of America; Universiti Teknologi Malaysia - Main Campus Skudai: Universiti Teknologi Malaysia, MALAYSIA

## Abstract

**Purpose:**

Multi-room proton therapy centers face unique operational challenges related to shared beam delivery systems, extended treatment hours, and high clinical complexity. This study presents a structured framework of key performance indicators (KPIs) and reports longitudinal single-institution operational reference data from a multi-room proton therapy center to support operational assessment and continuous quality improvement.

**Methods and materials:**

Operational KPIs were retrospectively evaluated for calendar year 2024 at a three-room proton therapy center utilizing pencil beam scanning. KPIs were categorized into three areas: beam performance, treatment planning and pre-treatment workflow, and treatment room operations. Beam performance metrics included site- and room-specific uptime and beam pauses. Treatment planning metrics quantified CT simulation–to–patient scheduling timelines with attribution to physician, dosimetry, and physics components. Treatment room operations metrics included appointment variance, slot utilization, and waiting-for-beam (WFB) time. Data were collected automatically using a commercial analytics platform integrating radio-frequency identification tracking and oncology information system. Metrics were analyzed longitudinally and stratified by treatment room, disease site, and workflow component.

**Results:**

For calendar year 2024, aggregate site uptime was 95.3%, weighted according to clinical operating time across the three treatment rooms; individual room uptimes ranged from 91.8% to 97.2%. Beam pauses were frequent but brief, resulting in 2,238 minutes of cumulative lost treatment time across 2024, equivalent to approximately 0.5% of total clinical operating time. Annual mean treatment planning time was 8.5 business days, with physician-related activities contributing the greatest variability across disease sites. Monthly average appointment variance ranged from 5 to 11 minutes, with most treatments initiated within 10 minutes of scheduled time. Across 2024, slot utilization was stable, with 73% of fractions completed within scheduled slots. Mean overrun and underrun durations were modest, although P90 overrun durations exceeded 10 minutes throughout the year. Mean WFB times were low and consistent, ranging from 1.5 to 2.3 minutes.

**Conclusions:**

This study presents a practical KPI framework and a single-institution operational reference data for multi-room proton therapy center. These metrics may support longitudinal monitoring, identification of workflow inefficiencies, data-driven decision-making, and future multi-institutional benchmarking efforts.

## Introduction

In 2024 nearly 50 proton therapy (PT) [1] centers were operational in the United States. While PT centers share many logistical challenges with traditional radiation oncology facilities, several unique factors significantly impact their workflow and resource utilization. Most PT centers utilize extended operating hours, often spanning from early morning until late evening. Furthermore, multi-room configurations frequently rely on a shared beam delivery system, creating operational dependencies that can compromise efficiency. PT facilities also typically manage a higher volume of complex cases, such as pediatric patients requiring anesthesia or re-irradiation. Finally, the inherent technical complexity of PT equipment increases the risk of downtime; the specialized nature of these components often requires vendor-specific intervention and may involve parts with limited onsite availability, occasionally leading to prolonged service interruptions.

Compared with conventional linac-based photon radiotherapy, proton therapy operations are still evolving toward comparable levels of operational robustness and standardization. In this manuscript, we present a set of key performance indicators (KPIs) designed to guide the operational assessment and optimization of a proton therapy facility. These KPIs are based on our experience at the Fred Hutchinson Cancer Center Proton Therapy Center (FHCC-PT), where operational data are collected automatically using a commercially available analytics platform interfaced with both the oncology-information-system (OIS) and the proton therapy delivery system.

Prior PTCOG publications and AAPM task group reports have addressed important aspects of proton therapy implementation, including the clinical and technical evolution of charged particle therapy [2], commissioning of intensity-modulated proton therapy systems [3], comprehensive proton therapy machine quality assurance [4], safety considerations in particle therapy [5], and QA efficiency in particle therapy [6]. These reports provide essential guidance for safe, accurate, efficient, and consistent proton therapy delivery; however, they do not establish longitudinal operational KPI reference data for multi-room PBS centers. A small number of proton therapy studies have reported data or methodological frameworks for selected KPIs, including system availability and downtime [7–9], proton beam-wait times [10,11], and patient treatment-slot duration [8,12,13]. However, several limitations remain. Many studies focus on a narrow subset of KPIs and do not address other operational metrics critical to modern proton therapy workflows, which are examined in this manuscript. Several studies are more than a decade old and reflect earlier generations of proton therapy systems using uniform scanning or double scattering [7,9], which differ substantially from modern multi-room pencil beam scanning centers. Even within conventional photon radiotherapy, publicly available data describing operational KPIs remain limited. Some investigations have evaluated time intervals from CT simulation to treatment initiation in photon therapy settings [14,15], where clinic hours, patient throughput, and treatment complexity may differ markedly from those of a dedicated proton center. Collectively, these limitations underscore the need for clearly defined, integrated KPI framework and longitudinal institutional reference data from modern multi-room pencil beam scanning proton therapy centers, which this study aims to address.

## Methods and materials

The KPIs evaluated in this study grew organically over time at FHCC-PT as part of routine operational monitoring and quality improvement. They were limited to metrics for which data are collected automatically through the clinical analytics platform and were selected based on their practical relevance to day-to-day operational decision-making. KPI selection was guided by three primary considerations: relevance to common operational challenges in a multi-room PBS proton therapy center, availability of automatically captured data, and ability to inform actionable operational decisions. These KPIs are grouped into three operational domains: Beam Performance, Planning and Pre-Treatment Workflow, and Treatment Room Operations. Beam performance metrics were included because shared accelerator and beamline performance directly affects available treatment capacity. Planning and pre-treatment workflow metrics were included because delays between CT simulation, plan approval, and treatment scheduling affect treatment start timeliness and resource allocation. Treatment room operations metrics were included because appointment variance, slot utilization, and waiting-for-beam time provide insight into daily schedule performance, room workflow, and shared-beam coordination. Table 1 summarizes the selected KPIs, their primary operational drivers, and the anticipated clinical and administrative impact associated with each KPI. The following sections provide detailed definitions of the KPIs and describe the methodology used to calculate and analyze them.

**Table 1:** Key operational KPIs and associated drivers in use at FHCC-PT.

Domain	Indicator	Measure (Operational Definition)	Primary Drivers	Operational Impact
**Beam Performance**	Uptime	Operational uptime rate for proton treatment rooms (%)	Equipment performance, Facilities infrastructure	Available treatment hours, staffing requirements, schedule predictability, and treatment delays
	Beam Pauses	Frequency and cumulative duration of beam pauses per treatment day		Treatment delays, overtime risk, staff workload, and treatment-day flow
**Planning &** **Pre-Treatment** **Workflow**	CT-Sim → Patient Scheduling(by Site)	Planning lead time from CT simulation to first fraction, stratified by anatomic site	Anatomic site, Physician, Physics, Dosimetry and RTT workflows	Schedule predictability, staffing load, and treatment start delays
	CT-Sim → Patient Scheduling(by Group)	Planning lead time stratified by treatment group (e.g., MD, Dosimetry, Physics)		
**Treatment Room Operations**	Appointment Variance	Deviation between scheduled appointment time and patient room entry time	RTT workflow, patient flow	Appointment timeliness, patient wait time, and downstream schedule compression
	Slot Utilization	Slot time variance (actual vs scheduled treatment duration)	RTT workflow, beam sharing, case complexity	Schedule stability, overtime risk, and throughput
	Waiting-for-Beam	Time from site setup completion to first beam delivery	Beam sharing, equipment performance	Slot utilization, appointment variance, and treatment hours

### Beam performance

#### Fred Hutchinson Cancer Center Proton Therapy facility.

The FHCC-PT is the only proton therapy facility in the Pacific Northwest region of the United States. The center operates three proton therapy treatment rooms [16] with distinct beam delivery configurations: one full 360° rotating gantry (GBR), one fixed horizontal beamline at 90° (FBR), and one inclined beamline room (IBR). The IBR room provides two beam delivery options: a horizontal beam and an inclined beam at 30° from vertical.

The availability of three treatment rooms with distinct geometric capabilities enables preferential patient assignment based on clinical indication and technical requirements at FHCC-PT. FBR, which is limited to a horizontal beam orientation, is primarily used for prostate treatments, unilateral head-and-neck cases, and select ocular and intracranial treatments delivered in a seated position. The GBR provides the greatest beam angle flexibility and is therefore reserved for pediatric patients and other complex cases requiring posterior–anterior or oblique beam arrangements. The IBR supports a broad range of disease sites, including breast, unilateral head-and-neck, liver, lung, esophagus, rectum, lymphoma, and intracranial treatments.

In addition, selected patients may be treated in a seated position [17], which offers operational advantages given that two of the three treatment rooms do not provide full gantry rotation. All treatment rooms utilize pencil beam scanning delivery [18], and patient positioning in 2024 was performed using orthogonal two-dimensional kV X-ray imaging.

Treatment planning was performed using the RayStation pencil beam scanning treatment planning system (RaySearch Laboratories, Stockholm, Sweden) [19], including the RayOcular [20] module for ocular treatments [21]. Additional clinical software included MIM (MIM Software Inc., Cleveland, OH, USA) for image manipulation and target contouring, and Limbus AI (Limbus AI Inc., San Francisco, CA, USA) for automated contour generation.

The FHCC-PT site is one of several radiation oncology sites within the Fred Hutchinson–University of Washington system. At FHCC-PT, dosimetrists, physics faculty, and radiation therapists are largely dedicated exclusively to proton therapy, while physician faculty rotate among the four affiliated sites. As a result, physicians may spend only a limited number of days at the proton center and perform the majority of contouring and plan review activities remotely.

FHCC-PT utilizes the Vantage platform (Transeo Radiotherapy Solutions, San Francisco, CA), which aggregates data from three primary sources: radio-frequency identification (RFID)-based patient badge tracking records, a live data feed from the vendor’s universal monitoring interface (UMI), and the OIS Mosaiq (Elekta Solutions AB, Stockholm, Sweden) database. Vantage collects and aggregates operational data from these systems and provides a suite of standard and custom applications for performance monitoring and analysis. The platform also includes a clinical whiteboard function that tracks treatment planning and delivery milestones and generates automated notifications and reminders throughout the patient workflow. For this study, the majority of KPI data were extracted from standard and custom Vantage applications. All analyses were performed using de-identified, aggregated operational metrics without identifiable patient-level information.

#### Uptime calculations.

Uptime percentage is one of the most critical key performance indicators for a proton therapy center, as it directly affects operational efficiency, financial performance, staffing requirements, and staff and patient flow. Higher uptime is associated with greater operational predictability and a reduced need for mitigation strategies such as schedule compression, overtime staffing, or patient rescheduling. For many centers, uptime is also a contractual performance metric with the proton therapy vendor, often tied to financial incentives or penalties. The contractual definition of uptime, however, is governed by service agreements and may differ from the definitions most relevant for internal staffing and resource management. While contractual uptime definitions are typically confidential, clinics may independently define internal uptime metrics to support quality improvement and operational decision-making.

Clinical operating time was first defined separately for each treatment room on each treatment day. It began with the start of daily clinical operations, typically at 7:00 AM when daily quality assurance procedures were initiated, and ended with the final beam delivery in that room. Clinical operating time included the scheduled treatment period for each room and, when downtime extended the treatment day, the additional time required to complete the scheduled clinical workload. Because patient volume and physics QA needs vary by room and by day, the end of clinical operating time was not fixed across rooms. The final beam delivery may correspond to either patient treatment or physics-related activities, including patient-specific or periodic physics quality assurance.

Downtime events included interruptions related to proton system deficiencies or equipment failures, as well as events outside of vendor control, such as power outages, treatment couch collisions, or facilities-related issues, including fire alarms or water leaks. Uptime, in minutes, was calculated by subtracting all downtime intervals from the total clinical operating time. Facility-wide downtime events, including cyclotron- or accelerator-related interruptions, were counted as downtime for all affected rooms during the time that each room was clinically operational.

Room-specific uptime was calculated independently for each treatment room, FBR, IBR, and GBR as defined below. Monthly and annual room-specific uptime values were calculated by summing uptime minutes and clinical operating time across all applicable treatment days before calculating the final percentage.


$$\begin{array}{c} Room\;uptime\;(\%)=\;\frac{Room\;uptime\;(mins)}{Room\;clinical\;operating\;time\;(mins)} \end{array}$$

Site uptime was then calculated by aggregating room uptime minutes and room clinical operating time across the three clinical treatment rooms. Monthly and annual site uptime values were calculated using the same aggregation approach across the relevant treatment days and rooms. Because site uptime was calculated from summed room uptime minutes and summed room clinical operating time, it represents a clinical-operating-time weighted aggregate rather than a simple average of the three room-specific uptime percentages.


$$\begin{array}{c} Site\;uptime\;(\%)=\;\frac{\sum_{FBR,\;IBR,\;GBR}Room\;uptime\;(mins)}{\sum_{FBR,\;IBR,\;GBR}Room\;clinical\;operating\;time\;(mins)} \end{array}$$

#### Beam pauses.

Beam pauses are brief interruptions in beam delivery caused by system instabilities in the beamline, nozzle, or cyclotron and are an important contributor to treatment delivery efficiency in proton therapy. Individual pauses typically last seconds to tens of seconds and may have minimal impact when considered in isolation; however, their short duration and high frequency make them difficult to capture reliably using manual downtime logging, as documenting numerous brief events is operationally impractical. At our institution, beam pauses are automatically recorded using UMI data, enabling comprehensive capture of short interruptions that would otherwise be underreported. While isolated pauses may have limited effect, their cumulative impact over a treatment day can lead to measurable efficiency losses, prolonged treatments, and downstream scheduling effects.

Longitudinal monitoring of beam pauses is therefore important, as pause frequency and duration may increase following major maintenance activities, such as cyclotron interventions or beamline calibration, and serve as indicators of overall system stability and health.

### Treatment planning and pre-treatment workflow metrics

Treatment planning workflows spanning the interval from patient CT simulation to plan approval and initial patient scheduling are captured using the Vantage analytics platform. Fig 1 illustrates the overall planning and pre-treatment workflow. The platform records completion times for key patient workflow milestones based on required user interactions at predefined stages, enabling automated data capture. The system also generates automated notifications to prompt task initiation. For example, when a dosimetrist creates a planning session and initiates a target contouring request, an automated notification is sent to the attending physician to begin contouring. By recording timestamped milestone completions, the system enables quantification of the time intervals associated with each workflow step. For example, the interval from a physician’s contouring request to contour completion is attributed to the physician component of the workflow time. Similar time intervals are defined for all participating clinical groups, as shown in Fig 1.

**Fig 1:**
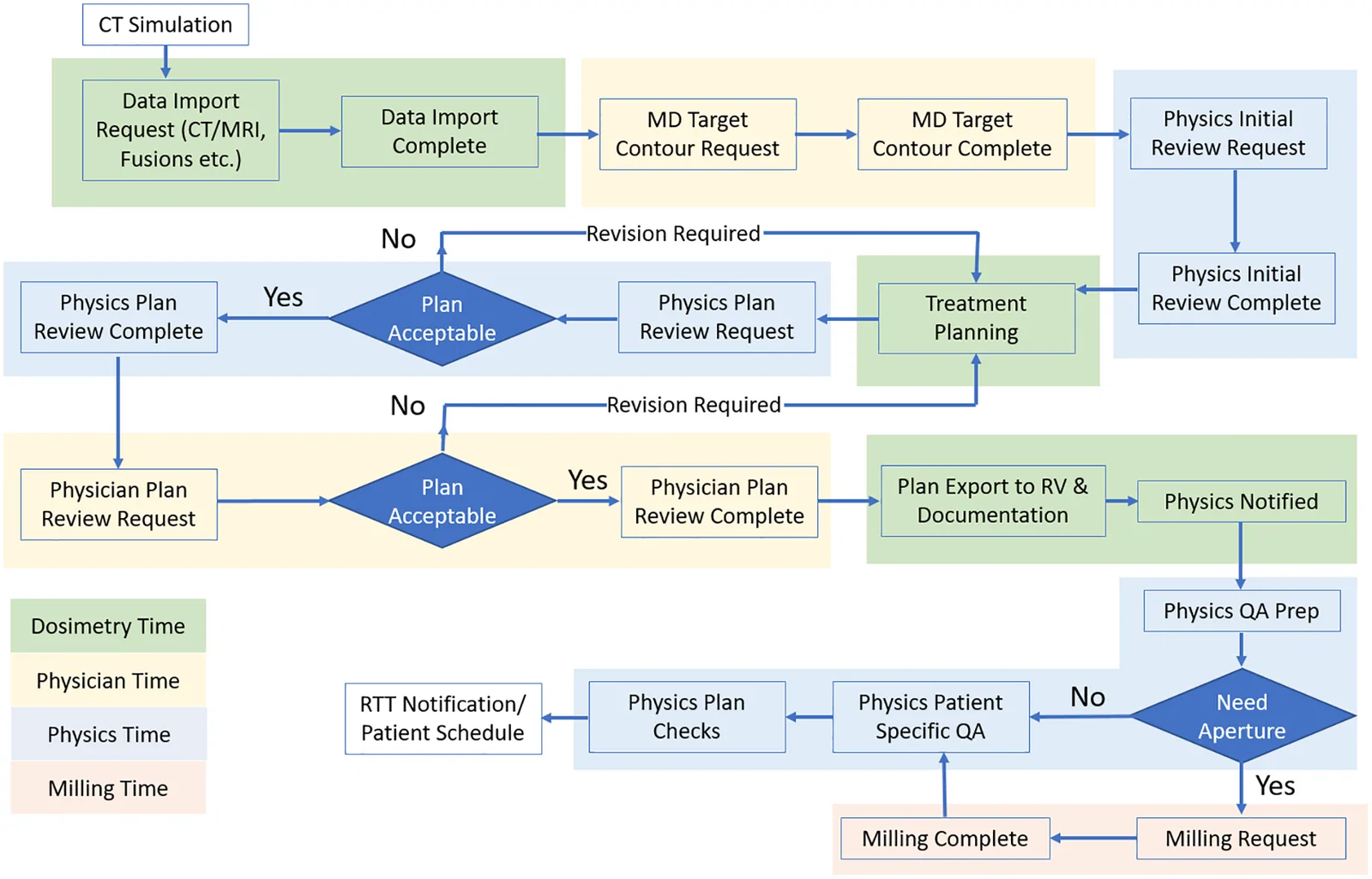
Treatment planning and pre-treatment workflow from CT simulation-to-patient scheduling. The diagram illustrates key workflow milestones, review steps, and decision points across dosimetry, physician, physics, and milling processes. Shaded regions indicate how workflow intervals were attributed to dosimetry time, physician time, physics time, and milling time for planning-time KPI analysis.

The CT simulation–to–patient scheduling interval can be evaluated across multiple dimensions. At our institution, two primary analyses are performed: planning duration stratified by anatomic site and by contributing clinical groups. For anatomic site–based analysis, the total planning duration is evaluated to identify systematic differences across disease sites.

### Treatment room operations

#### Appointment variance.

Appointment variance was defined as the difference, in minutes, between the scheduled appointment time and the actual time the patient entered the treatment room. This metric was derived from RFID data captured by antennas at the treatment room entrance, synchronized with beam-on events identified from the UMI data stream, and followed by a subsequent RFID trigger when the patient exited the room. For patients who entered the treatment room earlier than their scheduled time, the appointment variance was set to 0. Data points affected by treatment interruptions or downtime events outside of the control of the staff were also excluded to avoid confounding operational delays unrelated to patient flow.

Individual appointment variance values were aggregated monthly to calculate the mean appointment variance. The data were further stratified by treatment room and categorized into predefined time-interval bins to evaluate trends and room-specific differences in appointment variance.

#### Slot utilization.

Slot utilization quantifies the difference between the scheduled appointment slot duration and the actual time a patient occupies the treatment room. Patient in-room time is derived from RFID data, while the scheduled slot duration is obtained from the OIS. For optimal operational performance, this variance should be close to zero, indicating appropriate alignment between scheduled and delivered treatment times. Slots that are substantially longer than required waste capacity and underutilize clinical resources, whereas slots that are shorter than the time needed for efficient treatment delivery can lead to cumulative delays, increased appointment variance, and downstream schedule compression.

Key drivers of slot utilization include patient setup efficiency, image-guidance workflows, beam-sharing dynamics in a multi-room environment, staff training and experience, and beam-delivery performance. These factors collectively influence the duration of individual treatment fractions and the stability of the daily treatment schedule.

Slot utilization variance was evaluated by categorizing each treatment fraction as an overrun (actual duration exceeding the scheduled slot) or an underrun (actual duration shorter than the scheduled slot). For each month, the percentages of treatment slots classified as overruns and underruns were calculated, along with the mean absolute magnitude of the slot-time variance for each category. To characterize tail behavior and operational risk, the 90th percentile (P90) of the overrun and underrun magnitude distributions was also computed monthly. Analyses excluded treatment slots impacted by equipment downtime events and patient arrivals occurring after the scheduled appointment time.

#### Waiting-for-beam time.

Waiting-for-beam (WFB) time was defined as the interval between completion of patient setup, readiness for treatment, and the initiation of the first beam delivery. In a multi-room proton therapy center, WFB represents a direct measure of shared-beam resource contention, independent of patient positioning efficiency or treatment delivery time. As such, WFB captures system-level operational constraints that are not reflected by slot utilization metrics alone. This metric is derived exclusively from UMI data, using system flags that indicate when a treatment room is secured and when beam delivery begins.

WFB performance was summarized using the mean and 90th percentile (P90) of the waiting time distribution, with P90 selected to quantify intermittent but operationally significant beam contention. Metrics were aggregated monthly to assess seasonal variation, and by hour of the treatment day to evaluate accumulation effects associated with concurrent room utilization. Analyses were performed with treatment rooms pooled to characterize system-level behavior, with room-specific results evaluated separately to confirm the absence of dominant room-level effects.

## Results

### Beam performance

#### Treatment room & site uptime.

Fig 2 shows the monthly uptime for each of the three treatment rooms and the overall site, along with the corresponding number of delivered fractions. The overall site uptime for year 2024 was 95.3%, with annual uptimes of 97.2%, 91.8%, and 96.5% for FBR, IBR, and GBR, respectively. FBR, IBR, and GBR experienced 2, 5, and 3 months, respectively, during which monthly uptime fell below 95%. Across the year, FBR and GBR demonstrated relatively stable performance with consistently high monthly uptime, whereas IBR showed greater month-to-month variability. Overall site uptime remained in the mid-to-high 90% range for most months, with modest reductions observed during late summer before recovery in the fall. The number of delivered fractions varied seasonally, with higher clinical throughput observed in the spring and fall and lower volumes during the summer months. Descriptively, months with higher delivered fraction counts did not consistently correspond to lower site uptime.

**Fig 2:**
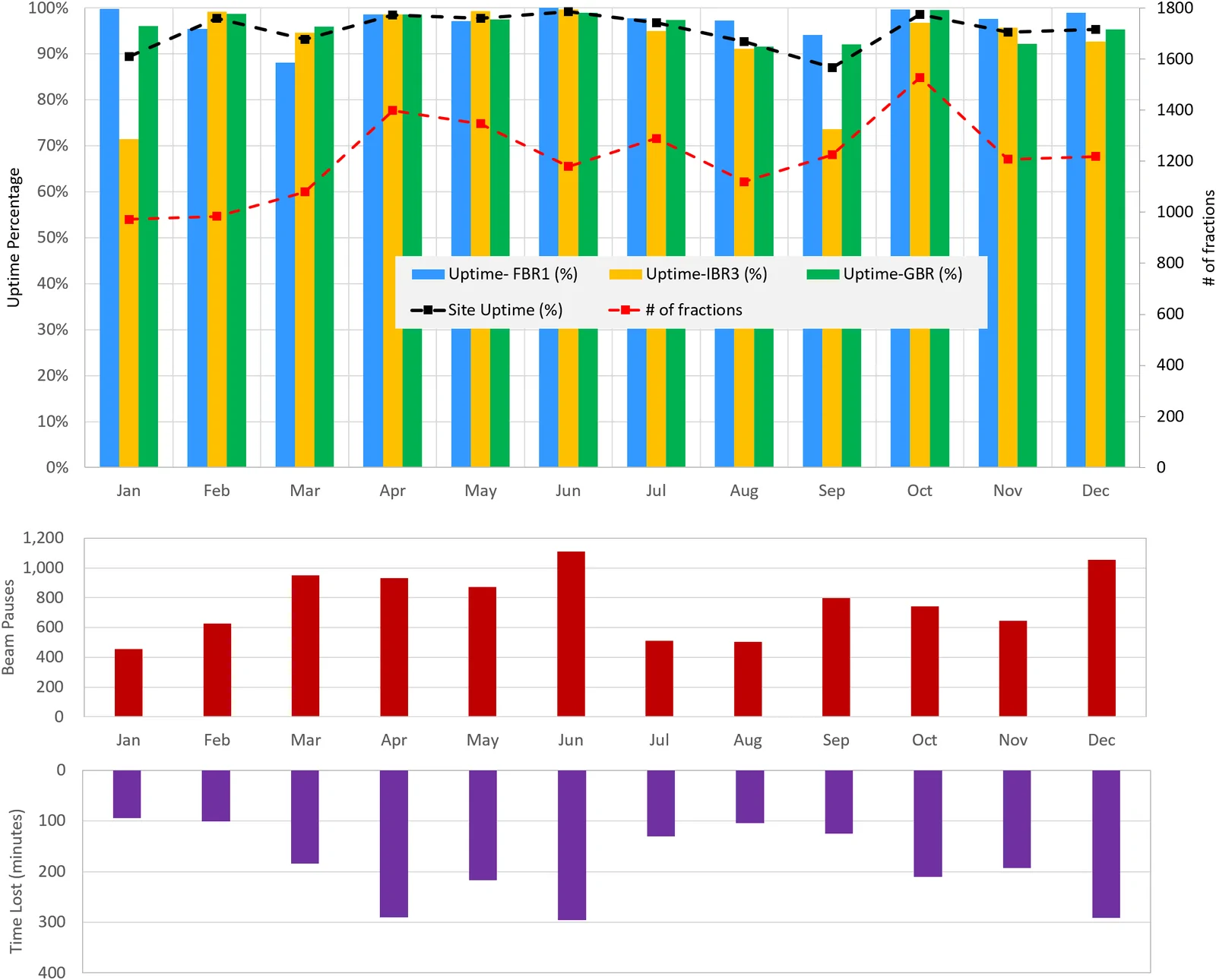
Monthly beam performance metrics for calendar year 2024. The top panel shows monthly uptime for each treatment room and aggregate site uptime, with the corresponding number of delivered treatment fractions shown on the secondary axis. The middle panel shows the total number of beam pauses recorded each month across all treatment rooms. The bottom panel shows the total monthly time lost due to beam pauses across all treatment rooms, plotted downward to emphasize cumulative time loss.

#### Beam pauses and time lost.

Fig 2 also shows monthly beam-pausing metrics aggregated across all treatment rooms, including the total number of beam pauses and the corresponding total time lost due to beam pauses. Across 2024, beam pauses resulted in 2,238 minutes, or 37.3 hours, of cumulative lost treatment time, corresponding to approximately 0.5% of total clinical operating time across the three treatment rooms. Monthly beam pauses demonstrated substantial month-to-month variability, ranging from approximately 450–1100 pauses per month, with corresponding monthly time lost ranging from approximately 95–295 minutes. Months with higher numbers of beam pauses generally corresponded to greater cumulative time lost, with the highest losses observed in April, June, and December. Although beam pauses can occur due to a variety of cyclotron, beamline, and room-specific system instabilities, temporary increases were observed following major cyclotron maintenance or intervention periods. A maintenance event near the end of February 2024 was followed by elevated beam pausing over subsequent months, during which the vendor implemented additional interventions and calibrations to improve system stability. Similarly, a cyclotron intervention at the beginning of September 2024 was followed by a transient increase in beam pausing. These findings demonstrate that beam pauses, although typically brief individually, can result in several hours of cumulative lost treatment time in selected months and may serve as a useful longitudinal indicator of system stability following major maintenance activities.

### Treatment planning and pre-treatment workflow metrics

Fig 3 summarizes the average time from CT simulation-to-patient scheduling, expressed in business days, with contributions from the physician (MD), dosimetry, and physics plan check, shown as a function of month and disease site**.** Across the year, total planning time remained relatively stable, typically ranging from approximately 7 to 9.5 business days, with modest month-to-month variation. Averaged over the year, the total planning time was 8.5 business days, with contributions of approximately 42% from MD time (contouring and plan review), 41% from dosimetry (treatment planning and documentation), and 17% from physics (patient-specific QA, physics reviews, and plan checks). Physics time was highly consistent throughout the year, ranging from 1.3 to 1.6 business days. MD time exhibited greater variability, potentially reflecting differences in case load, and ranged from 2.2 to 4.3 business days. Dosimetry time showed less variation than MD time, ranging from 2.9 to 3.7 business days. Months with higher case volumes, as indicated by the number of instances, did not demonstrate a consistent increase in total planning time.

**Fig 3:**
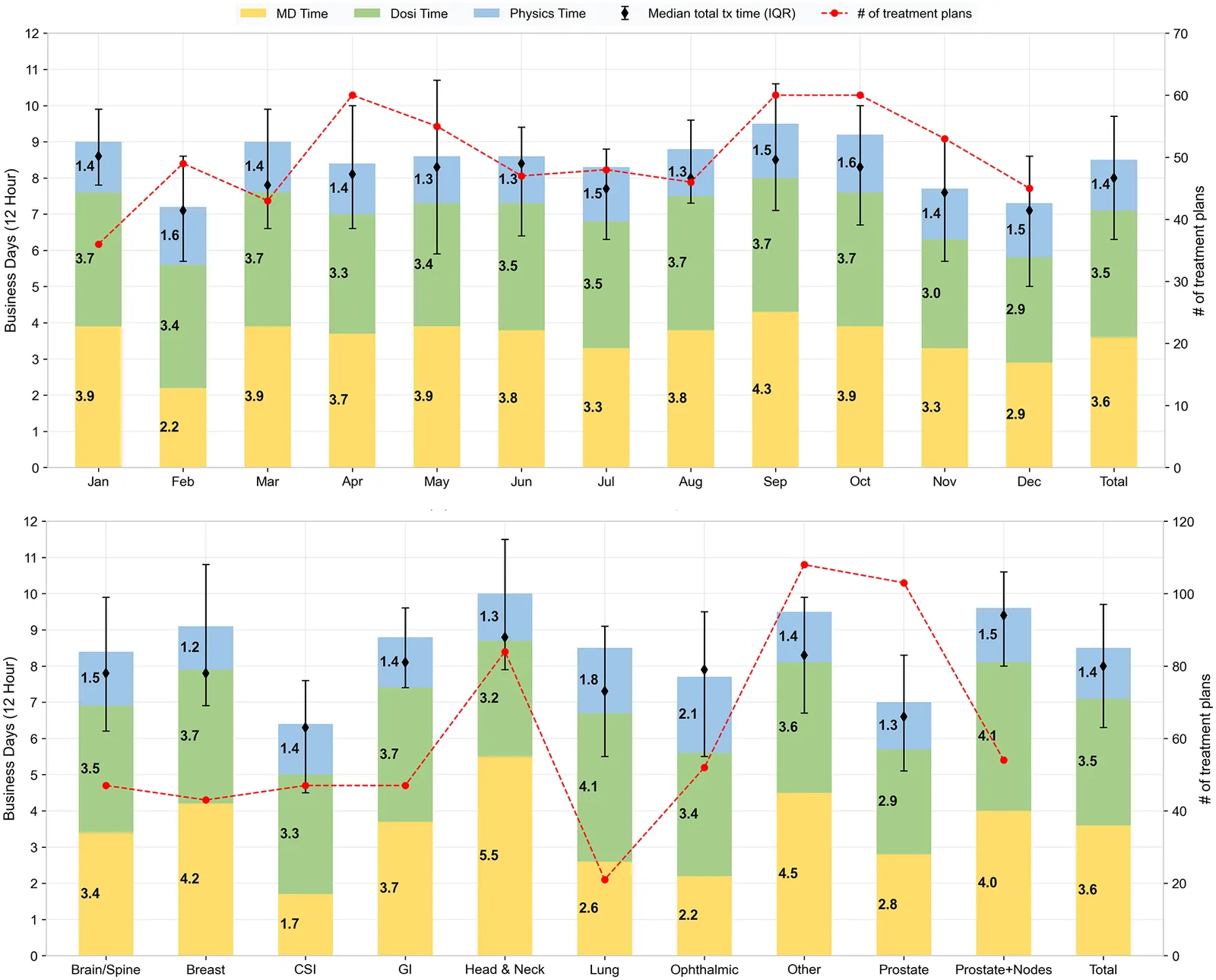
Treatment planning time for calendar year 2024, shown by month (top panel) and by disease site (bottom panel). Stacked bars represent the mean treatment planning time in business days, divided into physician (MD), dosimetry, and physics components. Black diamond markers indicate the median total planning time, and the vertical error bars represent the interquartile range (Q1–Q3) of the total planning time. The dashed line, shown on the secondary axis, indicates the number of treatment plans included in each group. The annual aggregate is shown as “Total” in the top and bottom panels.

When analyzed by disease site, total planning time ranged from 6.4 business days for CSI to 10.0 business days for head and neck cases. Physics time varied minimally across sites, ranging from 1.2 to 1.8 business days, except for ophthalmic cases (2.1 business days), for which beam-shaping aperture design and milling time were included in the physics component for simplicity. Dosimetry time was lowest for prostate cases (2.9 business days) and highest for lung and prostate with pelvic nodes (4.1 business days), reflecting increased planning complexity. The average dosimetry planning time across all disease sites was 3.5 business days. MD time demonstrated the greatest variability across disease sites, ranging from 1.7 business days for CSI cases to 5.5 business days for head and neck cases, with an overall average of 3.6 business days. These findings indicate that variability in overall planning timelines is primarily driven by the MD component and, to a lesser extent, by dosimetry time associated with case complexity.

### Treatment room operations

#### Appointment variance.

Fig 4 summarizes appointment variance as monthly average schedule variance with corresponding treatment volume (top panel) and the distribution of appointment variance by treatment room (bottom panel). Monthly average appointment variance ranged from approximately 5–11 minutes, with lower values earlier in the year and higher values toward the latter part of the year. The highest average variance occurred in September and October, coinciding with higher treatment volumes, while lower variance was observed in June and July. Descriptively, months with higher treatment volume did not consistently correspond to higher average appointment variance.

**Fig 4:**
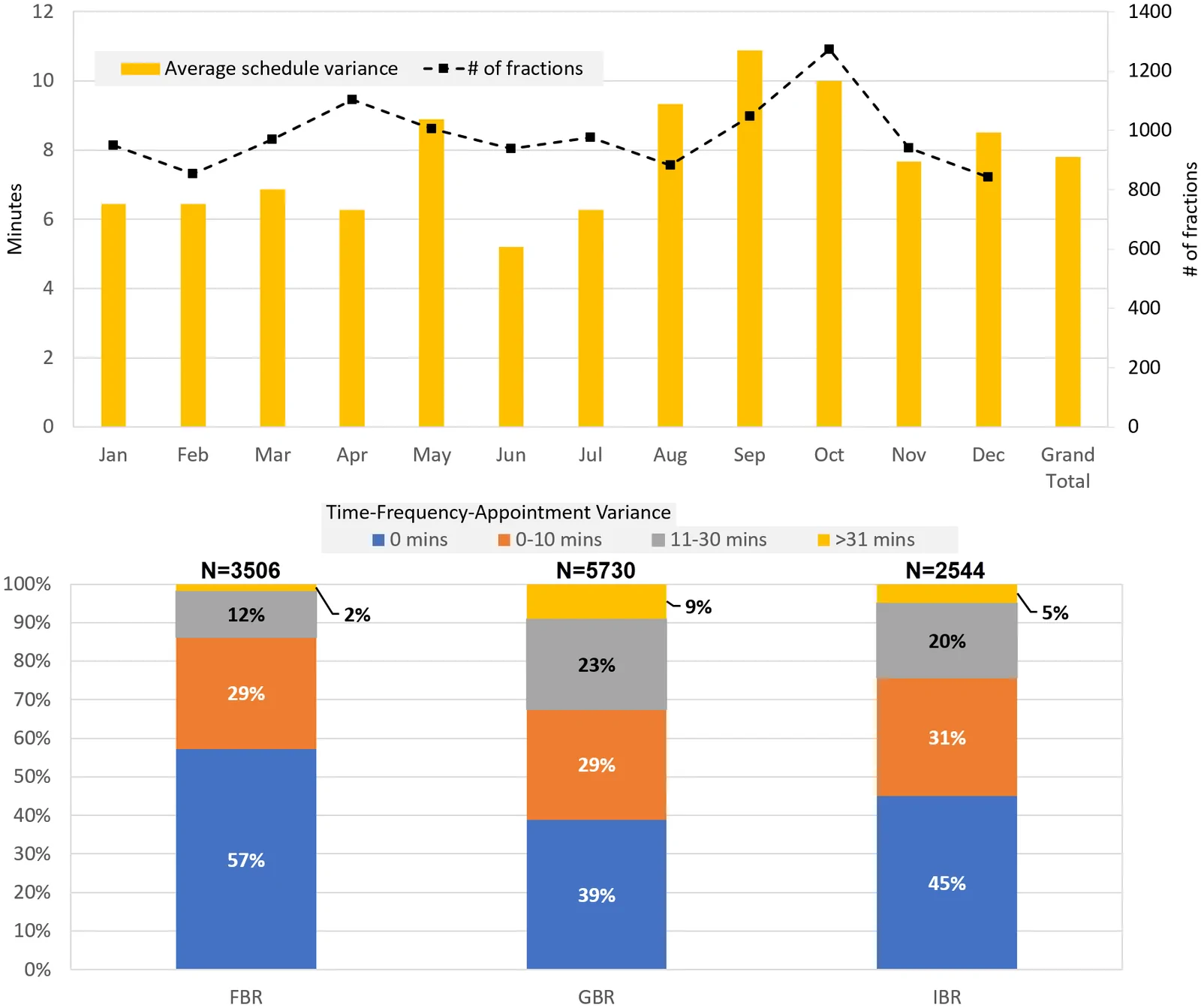
Appointment schedule variance performance by month and treatment room. The top panel shows the monthly average schedule variance (minutes) with the corresponding number of treatment fractions (secondary axis). The bottom panel shows the distribution of appointment variance by treatment room, categorized by delay intervals, with total instances indicated above each bar.

When stratified by treatment room, differences in appointment variance distributions were evident. FBR demonstrated the highest proportion of on-time treatments, with 57% of appointments occurring with zero variance and 86% initiated within 10 minutes of the scheduled time. Similarly, IBR showed 45% of appointments occurring on time, with over 76% initiated within a 10-minute window. GBR exhibited the greatest overall variability, with 39% of appointments occurring on time and fewer than 70% initiated within 10 minutes. These differences are partially attributable to variation in treatment complexity and clinical workload, with FBR predominantly treating simpler cases (e.g., prostate treatments with one to two beams), IBR treating moderately complex cases with a lower overall patient load, and GBR managing a higher patient volume with more complex treatments involving two to six beams. Across all treatment rooms, the majority of appointments were initiated within 10 minutes of the scheduled time, while larger delays (>30 minutes) constituted a small fraction of total treatments (approximately 6%). Overall, these results indicate that while average appointment variance remains modest, variability differs by treatment room, reflecting differences in workflow complexity and clinical throughput.

#### Slot utilization.

Fig 5 summarizes slot utilization performance as the proportion of fractions that under-ran or over-ran their scheduled appointment time and the corresponding magnitude of slot variance. Across the year, the majority of fractions were completed within the allocated slot, with under-runs occurring more frequently than over-runs in all months. Monthly under-run rates ranged from approximately 64% to 79%, whereas over-run rates ranged from approximately 21% to 36%. Periods with higher clinical volume, as reflected by the number of treatment instances, did not demonstrate a systematic shift toward increased overruns.

**Fig 5:**
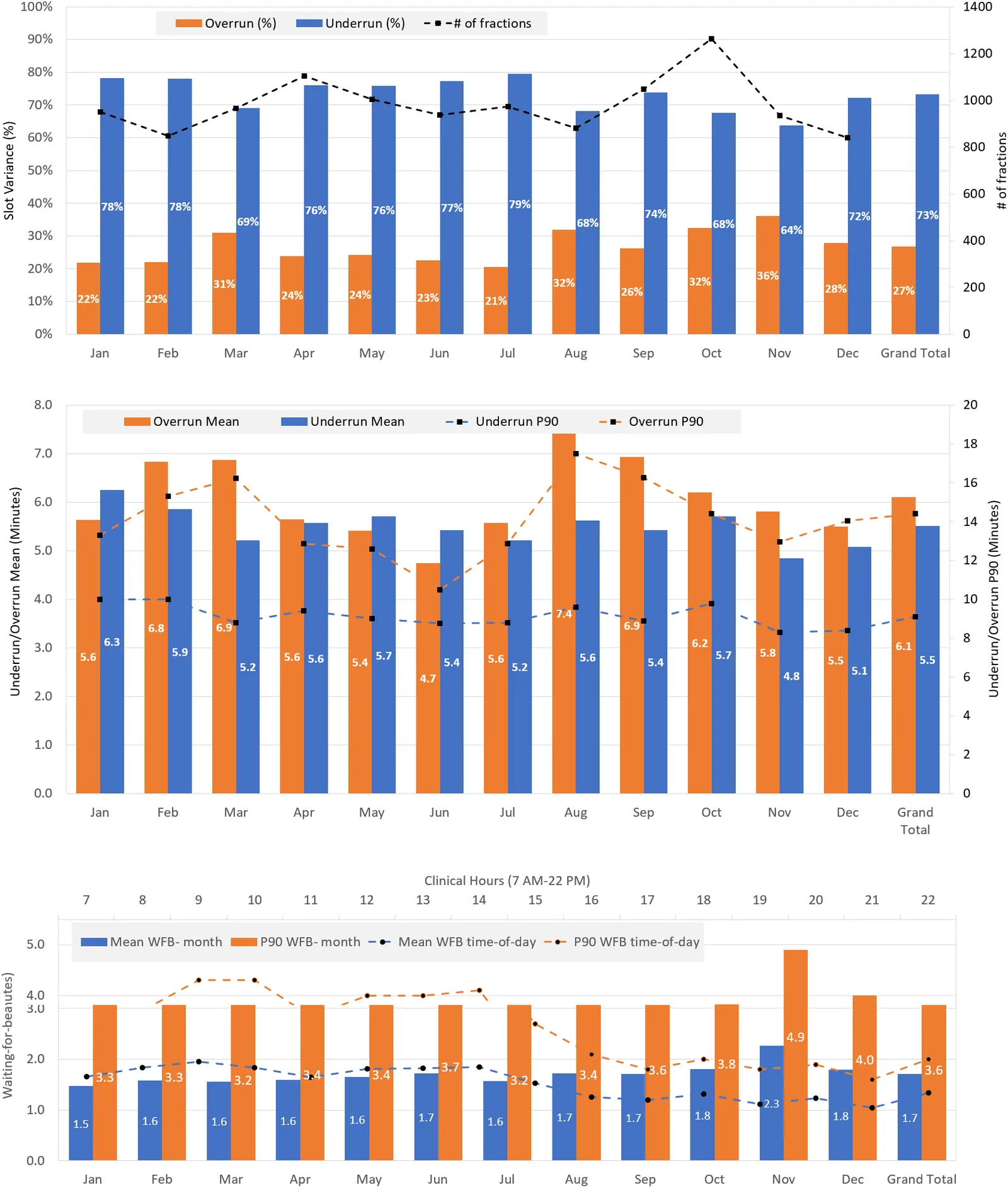
Slot utilization and waiting-for-beam performance for calendar year 2024. The top panel shows the proportion of fractions that under-ran or over-ran the scheduled appointment slot, with the number of treatment fractions overlaid (secondary axis). The middle panel shows the corresponding mean and P90 magnitudes of under-run and over-run durations. The bottom panel shows waiting-for-beam performance, presented as mean and P90 waiting times as a function of month (primary horizontal axis) and as a function of clinical hours (secondary horizontal axis).

The magnitude of slot variance showed modest month-to-month variation. Mean overrun durations ranged from approximately 5.4 to 7.4 minutes, whereas mean underrun durations ranged from approximately 4.8 to 6.3 minutes. The relatively narrow ranges of the monthly mean values suggest that average overrun and underrun magnitudes remained broadly consistent throughout the year. However, P90 overrun durations exceeded 10 minutes in all months, indicating that the upper tail of slot overruns was larger than suggested by the mean overrun values alone. P90 underrun durations were lower and more stable by comparison. These findings suggest that most deviations from scheduled slot duration were modest, but a subset of overruns may have contributed disproportionately to schedule compression and downstream delays.

#### Waiting-for-beam time.

WFB performance, summarized as mean and P90 waiting times and their corresponding time-of-day distributions during clinical hours, is shown in Fig 5. Mean monthly WFB times remained low and stable, ranging from approximately 1.5 to 2.3 minutes. P90 WFB times were consistently higher than the mean but remained below 5 minutes for all months, with only modest month-to-month variation. A relative increase in P90 WFB was observed in late fall, particularly in November, which may be attributed to vendor-cyclotron maintenance activities in early November that caused intermittent system instabilities.

Time-of-day analysis demonstrated a clear intraday pattern. Mean WFB times were higher during early morning clinical hours and decreased progressively throughout the day, reaching their lowest values in the late afternoon and early evening. A similar trend was observed for P90 WFB times, with greater variability in the morning and reduced waiting times later in the day. These patterns were consistent across months and are likely related to FBR and IBR completing treatments earlier due to lower patient volumes, thereby reducing competition for beam allocation to GBR later in the day.

## Discussion

This manuscript presents a selected set of KPIs relevant to multi-room proton therapy operations, which face technical and logistical challenges affecting clinical throughput, schedule predictability, staffing needs, and patient flow. While many KPIs can be defined, we focus on metrics routinely tracked and used at our clinic to guide operational improvements. These KPIs collectively capture factors influencing patient experience, including CT simulation–to–treatment intervals and day-to-day operations affected by uptime, room coordination, beam pauses, and shared-beam constraints.

Over the past several years, overall site uptime at our facility has ranged from 94.9% to 96.9%, comparable to the 96.9% uptime reported by Miller et al. [7] for a multi-room proton facility over a three-year period; however, their experience was based on uniform scanning with a fixed beam room and two 360° gantries. Suzuki et al. [8] reported a higher mean clinical availability of 98.5% for a single pencil beam scanning room over three years, but their analysis only included downtime events longer than 15 minutes and was limited to a single room. At our site, two major scheduled vendor maintenance periods occur annually, typically in February and November, are aligned with weekends or holidays, and require approximately 3–4 days of access, with one intervention often involving cyclotron opening for preventive maintenance. Based on multi-year operational experience, the post-maintenance period may be associated with an increased frequency of downtime events or beam instabilities, either globally or at the room level; for example, in 2024, IBR demonstrated increased beam pauses following the November maintenance. Access to longitudinal operational data such as these enables clinics to anticipate such trends and proactively develop mitigation strategies in collaboration with the vendor.

Beam pause analysis demonstrates that, while the average number and duration of pauses per fraction may appear modest, their cumulative impact can be substantial, resulting in several hours of lost treatment time per month. Beyond throughput implications, frequent beam pauses also affect RTT workload and staff and patient satisfaction. Periods of elevated beam pausing—particularly following major vendor interventions—can create increased time pressure and operational stress for RTTs, underscoring the importance of tracking this metric alongside uptime.

CT simulation–to–patient scheduling timelines, analyzed by month and stratified by anatomic treatment site, provide insight into workflow bottlenecks that may limit patient throughput. These data have informed institutional scheduling practices at our center, where a target turnaround time of 10 days has been established for standard (non-urgent) treatment plans. This timeline remains flexible for medically expedited starts. Even in expedited cases, decomposing timelines into physics, dosimetry, and physician components provides valuable insight into where efficiencies can be gained and which steps most strongly influence overall turnaround time.

Slot variance, defined as the difference between the scheduled appointment time and the actual room entry time, is used to assess day-to-day schedule adherence. The institutional goal at FHCC-PT is to maintain an average schedule variance of less than 10 minutes. In 2024, this threshold was exceeded only in September, coinciding with an increase in patient load in GBR—the sole full-gantry room at our facility, reserved for complex treatments requiring multiple beams. In 2024, up to 32% of treatments in GBR exceeded the 10-minute variance goal, compared with 14% and 25% in FBR and IBR, respectively, highlighting the disproportionate influence of complex case mix on this KPI.

Slot utilization provides complementary information by comparing allocated appointment duration with actual treatment time. At our clinic, the majority of appointment slots are 10 minutes (e.g., single-field prostate), 15 minutes (e.g., breast or two-field prostate), 20 minutes (e.g., head and neck, prostate with pelvic nodes), or 30 minutes (e.g., pediatric anesthesia and craniospinal irradiation). Across calendar year 2024, 73% of treatments were completed within the scheduled slot, with an average underrun of 5.5 minutes. The remaining 27% of treatments exceeded their allocated slot, with an average overrun of 6.1 minutes. Although mean overrun and underrun durations were modest, P90 overrun durations exceeded 10 minutes throughout the year, indicating that a subset of overrunning treatments had a disproportionate effect on slot-utilization performance. Slot durations can be adjusted to balance underrun and overrun rates within the clinic’s tolerance. At our institution, the operational goal is to maintain both P90 underrun and P90 overrun durations below 10 minutes; therefore, the 2024 data suggest that overrun performance may be an area for further optimization, even though average overrun durations remained relatively low.

WFB data demonstrated stable mean wait times throughout 2024, ranging from approximately 1.5 to 2.3 minutes. Although no formal institutional target has been established for this KPI, month-to-month variation is routinely monitored to ensure efficient beam operations. For instance, a relative increase in mean WFB duration, coincident with elevated beam pausing in IBR during November, prompted escalation to the beam vendor and subsequent system adjustments.

The KPIs evaluated in this study differ in their sensitivity to operational change. Beam-performance metrics, including uptime and beam pauses, are primarily sensitive to accelerator, beamline, and vendor-service factors. Planning workflow metrics are more sensitive to staffing models, physician availability, disease-site complexity, and clinic processes. Treatment-room operations metrics, including slot variance, slot utilization, and WFB time, are more directly affected by scheduling practices, RTT workflow, room-specific case mix, and shared-beam coordination. For centers with limited analytics resources, KPI implementation may reasonably begin with automatically captured, high-impact metrics such as uptime, CT simulation–to–treatment start intervals, planning turnaround time, and treatment-room entry delays before expanding to more detailed workflow decomposition.

Several evolving clinical and technological factors may influence interpretation and generalizability of the reported KPIs. Recent implementation of surface-guided radiation therapy and volumetric in-room imaging at our center affects a subset of patients and may alter setup times, pre-treatment workflows, slot utilization, schedule variance, site uptime, and waiting-for-beam metrics. These changes highlight the need to interpret operational data within the context of evolving clinical practice. Additionally, our facility’s configuration of three treatment rooms with distinct geometric capabilities may differ from other proton centers, including multi-gantry or single-room facilities. Variations in room geometry, shared-beam constraints, inter-room dependencies, scheduling practices, data-capture methods, and KPI definitions may limit direct numerical comparisons of operational metrics across published studies. Therefore, the values reported in this study should be interpreted as single-institution operational reference data rather than universal benchmark values. Nonetheless, the analytical framework presented provides a practical reference for centers seeking to assess operational performance and relate technical metrics to patient experience, staffing needs, and workflow efficiency.

## Supporting information

S1 Data Data analysis. (XLSX)
